# Efficacy and safety of dexmedetomidine in adult patients undergoing bronchoscopy: A systematic review and meta-analysis

**DOI:** 10.12669/pjms.42.7.16559

**Published:** 2026-07

**Authors:** Xin Huang, Junshi Li, Liang Zhang, Yu Du, Hongbo Su, Jianfeng Zuo

**Affiliations:** 1 Xin Huang, Department of Anesthesia, Changxing People’s Hospital, Huzhou, Zhejiang Province313100, P.R. China; 2 Junshi Li, Department of Anesthesia, Changxing People’s Hospital, Huzhou, Zhejiang Province313100, P.R. China; 3 Liang Zhang, Department of Anesthesia, Changxing People’s Hospital, Huzhou, Zhejiang Province313100, P.R. China; 4 Yu Du, Department of Anesthesia, Changxing People’s Hospital, Huzhou, Zhejiang Province313100, P.R. China; 5 Hongbo Su, Department of Anesthesia, Changxing People’s Hospital, Huzhou, Zhejiang Province313100, P.R. China; 6 Jianfeng Zuo, Department of Anesthesia, Changxing People’s Hospital, Huzhou, Zhejiang Province313100, P.R. China

**Keywords:** Bronchoscopy, Dexmedetomidine, Midazolam, Propofol, Remimazolam, Sedation

## Abstract

**Objectives:**

Dexmedetomidine has been increasingly used as an alternative sedative agent during bronchoscopy, but its comparative efficacy and safety remain unclear. This systematic review and meta-analysis aimed to assess the effectiveness and safety of dexmedetomidine compared with other sedative agents in adult bronchoscopy.

**Methodology:**

PubMed, Embase, CENTRAL, and Scopus were searched up to 17 December 2025 for randomised controlled trials (RCTs) comparing dexmedetomidine with other sedatives. Procedural success was assessed descriptively, while procedural duration, satisfaction scores, safety and hemodynamic parameters were analyzed quantitatively in a random-effects model.

**Results:**

Seventeen RCTs were included. Qualitative synthesis showed mixed results for the adequacy of sedation between dexmedetomidine and midazolam or propofol. However, studies did note a tendency for better sedation with remimazolam as compared to dexmedetomidine. Compared with midazolam, dexmedetomidine significantly reduced the incidence of hypoxia, but was associated with higher risks of bradycardia and reduced risk of hypotension; no consistent differences were observed in procedure duration or rescue sedation. Compared with propofol, dexmedetomidine was associated with lower rates of hypoxia and hypertension but a higher incidence of bradycardia. Compared with remimazolam, dexmedetomidine was associated with lower patient satisfaction scores and higher rates of hypoxia, whereas other safety outcomes did not show any significant difference. Studies comparing dexmedetomidine with opioids were scarce.

**Conclusions:**

Evidence shows dexmedetomidine is an effective sedative for adult bronchoscopy, comparable to midazolam and propofol in sedation levels. It may reduce hypoxia but increases bradycardia. Compared with remimazolam, it seems to offer less favourable sedation with a higher risk of hypoxia. However, data was from a limited number of studies.

***Registration No.:*** PROSPERO (CRD420251242030).

## INTRODUCTION

Flexible bronchoscopy continues to be an important diagnostic and therapeutic tool in modern respiratory care. It is being increasingly employed for simple airway checks to advanced interventions.^1^ While it is minimally invasive, bronchoscopy often lead to patient discomfort, anxiety, coughing, and instability in cardiovascular and respiratory functions.^2^ Therefore, effective sedation is important for safety and success. Ideally, use of sedatives should reduce anxiety and ensure patient comfort without affecting spontaneous breathing, blood pressure and heart rate, allowing quick recovery after the procedure.^3^ Traditionally, sedative agents used for bronchoscopy are benzodiazepines, opioids, and propofol, either singularly or in combination. Several of these drugs have certain drawbacks, like dose-related respiratory depression, oxygen desaturation, hypotension, and prolonged recovery times.^3^–^5^ This limits their use in older patients, with lung problems, or limited cardiopulmonary capacity.^2^ Therefore, there is increasing interest in exploring alternative drugs that provide better balance between effectiveness and safety.

Dexmedetomidine, a highly selective α2-adrenergic agonist, has become a promising sedative agent for bronchoscopic procedures.^6^ Its pharmacologic profile includes sedative and anxiolytic effects similar to natural sleep. The drugs has minimal impact on respiratory drive, and also include inherent analgesic and sympatholytic properties.^7^ These characteristics make dexmedetomidine especially suitable for bronchoscopy, wherein maintaining patient cooperation and airway reflexes is of utmost importance. However, its use is not without drawbacks. There have been reports of bradycardia and hypotension, especially with higher loading doses or rapid administration.^8^ Over the past decade, multiple randomised controlled trials (RCTs) have assessed the use of dexmedetomidine for sedation during bronchoscopy, comparing it with various agents like midazolam, propofol, and opioids.^9^–^11^

While some studies have shown benefits in patient comfort, procedural conditions, or fewer respiratory adverse events, the results have not been consistent. Differences in study design, comparator drugs, dosing protocols, outcome measures, and procedural complexity have limited strong conclusions. Two systematic reviews have previously examined the safety and efficacy of dexmedetomidine in bronchoscopy. Guo et al.^12^ compared dexmedetomidine with multiple agents but included only nine RCTs in their review, limiting the strength of the evidence. Liang et al.^6^ synthesised evidence from six RCTs comparing dexmedetomidine with midazolam in adults, but it was published only as a letter and did not provide a comprehensive assessment of outcomes and evidence quality. Since the use of dexmedetomidine in bronchoscopy is gradually expanding, an updated systematic review and meta-analysis is necessary. This study aimed to assess the effectiveness and safety of intravenous dexmedetomidine compared with other sedatives during bronchoscopy by combining data from RCTs.

## METHODOLOGY

This systematic review and meta-analysis was conducted in accordance Cochrane guidelines.^13^ The review has been reported in accordance with the Preferred Reporting Items for Systematic Reviews and Meta-Analyses (PRISMA) statement.^14^ The review was also registered on PROSPERO (CRD420251242030).

### Literature search strategy:

Two independent reviewers searched the literature to identify all relevant RCTs. The search included the electronic databases of- PubMed, Embase, the Cochrane Central Register of Controlled Trials (CENTRAL), and Scopus. The search spanned from the inception of each database up to December 17, 2025. Details of the search strategy are shown in Supplementary Table-I. Supplementary searches were also performed in ClinicalTrials.gov and Google Scholar to identify ongoing or unpublished studies. Additionally, we also checked the reference lists of relevant reviews and included articles to identify any eligible studies not searched in the electronic searches.

**Supplementary Table-I:** Search strategy for the systematic review and meta-analysis.

***1. PubMed (MEDLINE)***
((“Bronchoscopy”[Mesh] OR bronchoscopy OR bronchoscopic OR endobronchial) AND (“Dexmedetomidine”[Mesh] OR dexmedetomidine) AND (sedation OR sedative OR anesthesia OR anaesthesia)) AND (randomized controlled trial OR randomized[Title/Abstract] OR randomised[Title/Abstract] OR randomly[Title/Abstract])
***2. Embase***
(‘bronchoscopy’/exp OR bronchoscopy OR bronchoscopic OR endobronchial) AND (‘dexmedetomidine’/exp OR dexmedetomidine) AND (sedation OR sedative OR anesthesia OR anaesthesia) AND (‘randomized controlled trial’/exp OR random*:ti,ab)
***3. Cochrane Central Register of Controlled Trials (CENTRAL)***
(bronchoscopy OR bronchoscopic OR endobronchial) AND (dexmedetomidine) AND (sedation OR anesthesia OR anaesthesia)
***4. Scopus***
TITLE-ABS-KEY (bronchoscopy OR bronchoscopic OR endobronchial) AND TITLE-ABS-KEY (dexmedetomidine) AND TITLE-ABS-KEY (sedation OR sedative OR anesthesia OR anaesthesia) AND TITLE-ABS-KEY (randomized OR randomised OR “controlled trial”).

### Eligibility criteria:

Studies were selected according to predefined inclusion and exclusion criteria based on the Population, Intervention, Comparator, and Study design framework.

### Inclusion criteria:

- RCTs only.
- Adult patients (≥18 years) undergoing flexible bronchoscopy or bronchoscopic interventions.
- Use of intravenous dexmedetomidine as the primary sedative agent.
- Comparison with other sedative regimens, including benzodiazepines, opioids, propofol, or alternative sedatives.
- Reporting of at least one outcome related to sedation efficacy, patient or operator satisfaction, rescue medication use, or adverse events.

### Exclusion criteria:

- Non-randomized or observational studies.
- Studies involving pediatric populations.
- Trials in which dexmedetomidine was not administered intravenously.
- Studies lacking a comparator group or using placebo.
- Studies using different drug combinations in the control group.
- Conference abstracts without sufficient data for extraction.

### Study selection:

All retrieved records were imported into a reference management system. Duplicate citations were removed. Two reviewers independently screened all the titles and abstracts to find potentially eligible studies. Full-text articles were then assessed for eligibility. Any disagreements during the study selection process were resolved through discussion involving a third reviewer.

### Data extraction:

Data extraction was independently conducted by two reviewers using a standardized form. We extracted information on study characteristics, patient demographics, sedation protocols, comparator regimens, rescue sedatives, sedation scales, and predefined outcomes. Continuous outcomes were recorded as means and standard deviations. When outcomes were reported as medians with interquartile ranges or ranges, these were converted to means and standard deviations with validated statistical methods.^15^ To ensure accuracy, all data were cross-checked by a third reviewer.

### Outcomes of interest:

The primary outcomes of interest included: sedation quality and satisfaction, like sedation scores, patient satisfaction scores, and bronchoscopist or anesthesiologist satisfaction scores. Secondary outcomes included the requirement for rescue sedative medications, procedural duration, and procedure-related adverse events, including hypoxia, hemodynamic instability (tachycardia, bradycardia, hypertension and hypotension).

### Risk of bias assessment:

Two reviewers independently assessed the methodological quality of the included RCTs using the Cochrane Risk of Bias 2 (RoB 2) tool.^13^ They evaluated the studies for the randomisation process, deviations from intended interventions, missing outcome data, outcome measurement, and the selection of reported results. Each domain was rated as low risk, some concerns, or high risk of bias. Any disagreements were resolved in consultation with the third reviewer.

### Statistical analysis:

Two reviewers compiled all outcome data, which were further examined by all authors. If any outcome was measured by differing scales across the included studies, only qualitative synthesis was conducted. When adequate and homogenous data was available, quantitative synthesis was carried out using ‘R’ software (meta and metaphor packages). Continuous outcomes were pooled using standardised mean differences (SMD) or mean differences (MD) with corresponding 95% confidence intervals (CI). Dichotomous outcomes were combined using odds ratios (OR). A random-effects meta-analysis model was used for all analyses. Separate analyses were conducted for each comparator. Due to the limited number of studies for each comparison, publication bias could not be assessed by funnel plots. Statistical heterogeneity was assessed using the I² statistic. Values >50% indicated high heterogeneity. Certainty of evidence of important outcomes was assessed using GRADE.

## RESULTS

**Fig.1:**
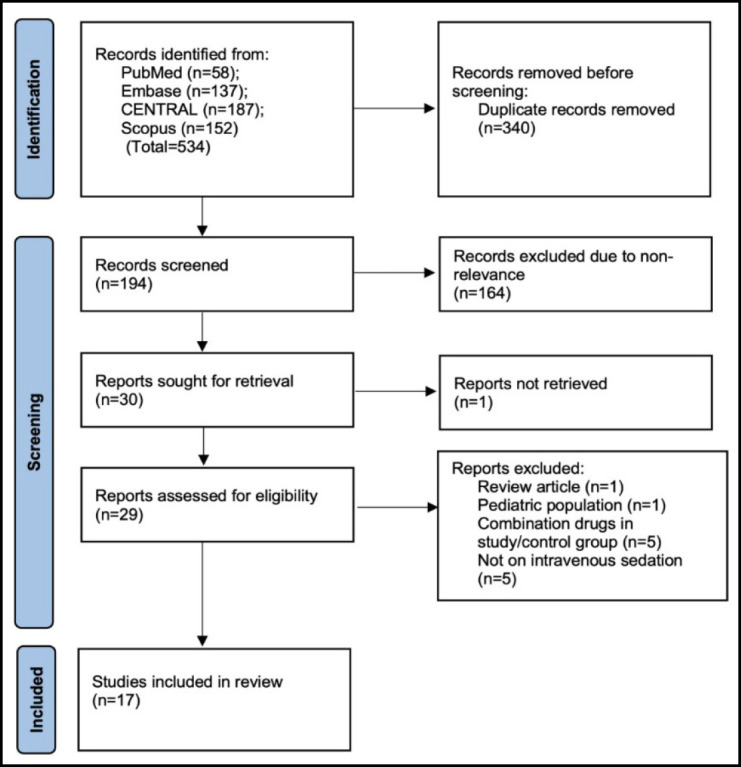
Study flowchart.

The study selection process is outlined in Fig.1. Seventeen RCTs met the inclusion criteria.^9^–^11^,^16^–^29^ The trials included were mainly conducted in Asia, with some studies from Israel and Lebanon (Table-I). Most studies were on flexible bronchoscopy, while two were on patients undergoing endobronchial ultrasound–guided transbronchial needle aspiration (EBUS-TBNA). Dexmedetomidine was compared to various sedatives or analgesics, like midazolam, propofol, remimazolam, opioids (fentanyl, remifentanil, or alfentanil), or combination regimens. The dosing was consistent across studies. Typically, a loading dose of 0.25–1.0 µg/kg was administered over 10–15 minutes, followed by a maintenance infusion of 0.2-1.4 µg/kg/h. Comparator drug doses varied across studies.

**Table-I:** Details of included studies.

Study ID Location	Procedure Type	Groups	Sample Size	Age (years)	Male (%)	ASA III (%)	Dexmed dose	Comparator drug dose	Rescue drug	Sedation scale
Chen 2024 China	Flexible bronchoscopy	Dexmed	30	49 ± 10	80	6.7	0.6 µg/kg loading over 10 min	Remimazolam 0.073 mg/kg or 0.093 mg/kg, respectively for 10 min	Propofol	MOAA/S
		Control-1	29	46.6 ± 14.4	60	3.4				
		Control-2	30	43.3± 15.3	76.7	3				
Xu 2024 China	Flexible bronchoscopy	Dexmed	60	60.9 ± 4.2	68.3	0	0.5 µg/kg over 10 min + 0.2–0.7 µg/kg/h	Remimazolam 6 mg/kg/h induction + 1–2 mg/kg/h maintenance	Propofol	MOAA/S
		Control	60	60.7 ± 4.3	66.7	0				
Zhou 2024 China	Flexible fiberoptic bronchoscopy	Dexmed	182	61 (55–65)	63.7	4.4	0.1–0.2 mL/kg loading + 0.1–0.5 mL/kg/h	Remimazolam 0.1–0.2 mL/kg loading + 0.1–0.5 mL/kg/h	Propofol	MOAA/S
		Control	182	60 (52–66)	69.2	7.7				
Chen 2022 China	Flexible bronchoscopy	Dexmed	73	56.1 ± 6	72.6	NR	0.5 µg/kg loading over 10 min + 0.2–0.7 µg/kg/h	Remimazolam 12 mg/kg/h induction + 1–2 mg/kg/h maintenance	Propofol	NR
		Control	73	57.1 ± 5.6	75.3					
Pertzov 2022 Israel	Flexible bronchoscopy	Dexmed	30	58.8 ± 15.1	43.3	NR	1 µg/kg over 15 min + 0.5 µg/kg/h	Propofol 0.5–1 mg/kg induction + 100–200 µg/kg/min infusion	Propofol	Richmond Agitation Sedation Scale
		Control	33	62.9 ± 9.7	69.7					
Chouhan 2023 India	Flexible bronchoscopy	Dexmed	40	52.3 ± 14.3	55	7.5	1 µg/kg over 10 min	Midazolam 0.05 mg/kg Propofol 0.5 mg/kg induction	Propofol	Composite score
		Control-M	40	58.1 ± 8.3	67.5	7.5				
		Control-P	40	55.6 ± 11.2	60	12.5				
Kim 2021 Korea	Flexible bronchoscopy	Dexmed	48	63.7 ± 11.3	68.8	NR	0.25µg/kg loading over 10 min + 0.25-0.75µg/kg/h	Midazolam 0.05 mg/kg loading + 1mg boluses	Midazolam	Ramsay Sedation Scale
		Control	54	63.9 ± 11.0	72.2					
Kumari 2021 India	EBUS-TBNA	Dexmed	99	44 ± 14	56.6	0	1 µg/kg loading + 0.6 µg/kg/h infusion	Midazolam 2 mg bolus	Midazolam	Ramsay Sedation Scale
		Control	98	49 ± 14	51					
Magazine 2021 India	Flexible bronchoscopy	Dexmed	24	48.5 ± 13.1	54.2	NR	0.75 µg/kg over 10 min	Midazolam 0.035 mg/kg	Midazolam	Ramsay Sedation Scale
		Control	24	44.4 ± 12.0	41.7					
Yuan 2016 China	Flexible bronchoscopy	Dexmed	50	59.8 ± 7.6	54	30 28	1 µg/kg over 10 min + 0.5 µg/kg/h	Propofol (2–4 µg/mL)	Lidocaine	MOAA/S
		Control	50	60.5 ± 6.9	44					
Lin 2020 China	EBUS-TBNA	Dexmed	25	59.7 ± 14.1	56	NR	1 µg/kg over 10 min + 0.5–1.4 µg/kg/h	Propofol (2.0 µg/mL)	NR	MOAA/S
		Control	25	59.4 ± 12.2	52					
Magazine 2020 India	Flexible bronchoscopy	Dexmed	27	47.1 ± 13.4	70.4	NR	0.5 µg/kg over 10 min	Midazolam 0.035 mg/kg	Midazolam	Ramsay Sedation Scale
		Control	27	45 ± 14.3	51.9					
Liao 2012 China	Flexible bronchoscopy	Dexmed	99	58.5 ± 9.1	61.6	NR	Up to 1 µg/kg over 10 min + 0.5 µg/kg/h	Midazolam 2 mg bolus + 1 mg titrated	NR	Ramsay Sedation Scale
		Control	99	60.1 ± 8.4	63.3					
Ryu 2012 Korea	Flexible bronchoscopy	Dexmed	35	52.9 ± NR	57	NR	Infusion 1.0 ± 0.4 µg/kg/min	Remifentanil infusion	Airway maneuvers only	MOAA/S
		Control	35	52.9 ± NR	51					
Goneppanavar 2015 India	Flexible bronchoscopy	Dexmed	27	49.7 ± 13.9	74.1	NR	1 µg/kg over 10 min (single bolus)	Midazolam 0.02 mg/kg	Midazolam	Ramsay Sedation Scale
		Control	27	52.5 ± 15.1	88.9					
Riachy 2017 Lebanon	Flexible bronchoscopy	Dexmed	53	NR	58.5	NR	0.5 µg/kg over 10 min + lidocaine	Alfentanil 10 µg/kg bolus + lidocaine	Midazolam	Nursing Instrument for the Communication of Sedation score
		Control	55		43.6					
Zhang 2021 China	Flexible bronchoscopy	Dexmed	222	58 ± 14	62.6	NR	1 µg/kg (max 50 µg) + midazolam	Fentanyl 1 µg/kg (max 50 µg) + midazolam	Midazolam	Bispectral index
		Control	211	60.2 ± 11.6	59.7					

NR, not reported; ASA, American Society of Anesthesiologist; max, maximum; IV, intravenous; MOAA/S, Modified Observer’s Assessment of Alertness/Sedation; Dexmed, Dexmedetomidine; EBUS-TBNA, endobronchial ultrasound–guided transbronchial needle aspiration.

Propofol and midazolam were the common rescue sedatives used. Patients were mostly middle-aged to older adults. The percentage of male participants varied from around 40% to 80%. Sedation levels and procedural conditions were assessed using the Modified Observer’s Assessment of Alertness/Sedation (MOAA/S) and the Ramsay Sedation Scale. Other tools, such as the Richmond Agitation–Sedation Scale, bispectral index monitoring, or combined scores, were also employed in individual trials. Several studies reported procedural success or sedation adequacy. However, quantitative synthesis was limited by variations in the outcome definitions. A detailed descriptive analysis is shown in Table-II.

**Table-II:** Descriptive analysis of success of sedation.

Study ID	Comparator	Outcome	Result
Chen 2024	Remimazolam	1. Success rate of sedation, which needed the following requirements to be satisfied at the same time: a. collaborate to accomplish tracheal intubation; b. no rescue sedative; c. no rescue local anesthetic.	1. The success rates of sedation in groups remimazolam (0.093mg/kg) and dexmed were 93.3% and 86.7%, respectively, which were higher than that in remimazolam (0.073mg/kg) (58.6%) (p=0.002).
			2. Compared with both remimazolam groups, dexmed group had more patients with good intubation conditions. However, the outcome did not differ between the two remimazolam groups.
		2. Good intubation conditions were defined as intubation comfort score ≤ 2, cough score ≤ 1, and post-intubation score = 1.	
Xu 2024	Remimazolam	1. Incidence of intraoperative awareness	1. No statistical significant difference between remimazolam and dexmed
		2. Bronchoscopy feasibility (extent of coughing, vocal cords movements, and limb movements; scored 3 to 12 with 3 representing the optimal score and 12 the worst one)	
			2. Scores in remimazolam group significantly better at T2 (immediately after passing the bronchoscope next to the glottis) but not later.
Zhou 2024	Remimazolam	Interruption rate, defined as body movements or coughing, making the operating physicians suspend the operation for physical restraint or anesthesiologists administering rescue medication.	The interruption rate was 8.2 % in the remimazolam group and 39.2 % in the dexmed group (P < 0.001)
Chen 2022	Remimazolam	Successful procedure (Not defined)	Successful completion rate of the procedure was 94.52% in the remimazolam group and 91.78% in the dexmed group with no statistical significant difference.
Pertzov 2022	Propofol	NR	NR
Chouhan 2023	Midazolam Propofol	Composite score by consisting five different parameters, i.e., sedation, calmness, respiratory response, physical movement, and facial tension. Each parameter was given a score of 1–5. Ideal: 5–10; Acceptable: 11–15, and Unacceptable: >15	Mean composite score of dexmed group was ideal showing better patient tolerance and cooperation compared to midazolam and propofol groups.
Kim 2021	Midazolam	NR	NR
Kumari 2021	Midazolam	NR	NR
Magazine 2021	Midazolam	Composite score by consisting five different parameters, i.e., sedation, calmness, respiratory response, physical movement, and facial tension. Each parameter was given a score of 1–5. Ideal: 5–10; Acceptable: 11–15, and Unacceptable: >15	The composite score was in the ideal category in 24 patients in dexmedetomidine group and 21 in midazolam group, at nasopharynx (P=0.234). The corresponding values at the level of trachea were 23 and 16 (P=0.023).
Yuan 2016	Propofol	Cough and discomfort scores rated by bronchoscopist and patient	No statistical significant difference between the two groups
Lin 2020	Propofol	Wakefulness, hearing/seeing during sedation, and tolerance by patient	Patients in the dexmedetomidine group were more likely to report wakefulness , hearing something, seeing something than those in the propofol group. In terms of tolerance, patients in the dexmedetomidine group were more likely to perceive procedure-related symptoms.
Magazine 2020	Midazolam	Composite score by consisting five different parameters, i.e., sedation, calmness, respiratory response, physical movement, and facial tension. Each parameter was given a score of 1–5. Ideal: 5–10; Acceptable: 11–15, and Unacceptable: >15	Total composite score did not differ significantly between dexmedetomidine and midazolam group at nasopharynx or at the level of trachea.
Liao 2012	Midazolam	Discomfort score	No statistical significant difference between the two groups
Ryu 2012	Remifentanil	NR	NR
Goneppanavar 2015	Midazolam	Composite score by consisting five different parameters, i.e., sedation, calmness, respiratory response, physical movement, and facial tension. Each parameter was given a score of 1–5. Ideal: 5–10; Acceptable: 11–15, and Unacceptable: >15	The composite score was ideal or acceptable in 15 patients in midazolam group and 26 patients in dexmed group. The mean scores were 14.48 ±3.65 in midazolam group and 9.41 ±3.13 in dexmed group (p<0.001).
Riachy 2017	Alfentanil	Bronchoscopy score which was computed by combining threevariables graded 1–4: movement of the vocal cords, cough occurrence and limb movement. The final bronchoscopyscore varied between 3 and 12 where 3 represented the optimal score and 12 represented the worst score	No statistically significant differences in the mean bronchoscopy score were found between the groups.
Zhang 2021	Fentanyl	NR	NR

NR, not reported.

### Risk of bias assessment:

**Supplementary Fig.1:**
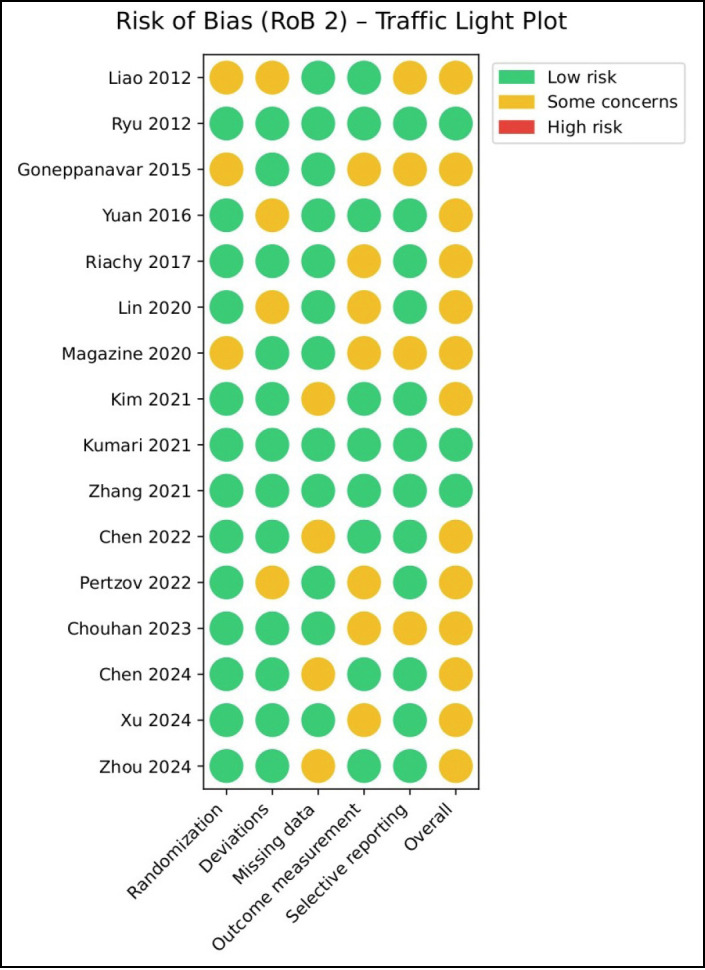
Traffic light figure for risk of bias analysis.

Risk of bias assessment as per the authors’ judgment, is shown in Supplementary Fig.1. Three trials had low risk of bias across all domains, while all other trials had some concerns in one or more domains.

### Dexmedetomidine versus Midazolam:

Several studies reported procedural success or sedation adequacy. However, quantitative synthesis was limited by variations in the outcome definitions. A detailed descriptive analysis is shown in Table-II. Chouhan et al.,^9^ assessed sedation success using a composite score that included sedation depth, calmness, respiratory response, physical movement, and facial tension. Lower scores indicating better tolerance. Their results showed that dexmedetomidine group achieved mean composite scores within the optimal range, indicating superior patient tolerance and cooperation compared to midazolam. Similarly, Magazine et al. (2021)^23^ used the same composite scoring system at various stages of the procedure. They noted that, at the level of the nasopharynx, the proportion of patients achieving an ideal composite score was similar between the dexmedetomidine and midazolam groups. However, at the tracheal level, a higher proportion of patients in the dexmedetomidine group achieved ideal scores. Conversely, another trial by Magazine et al. (2020)^21^ reported no significant difference in total composite scores between the two groups. Goneppanavar et al.^17^ utilised a similar composite score to evaluate procedural tolerance and noted better results with dexmedetomidine. Liao et al.^29^ assessed patient discomfort using a dedicated discomfort score and observed no statistically significant difference between the two groups.

**Supplementary Fig.2:**
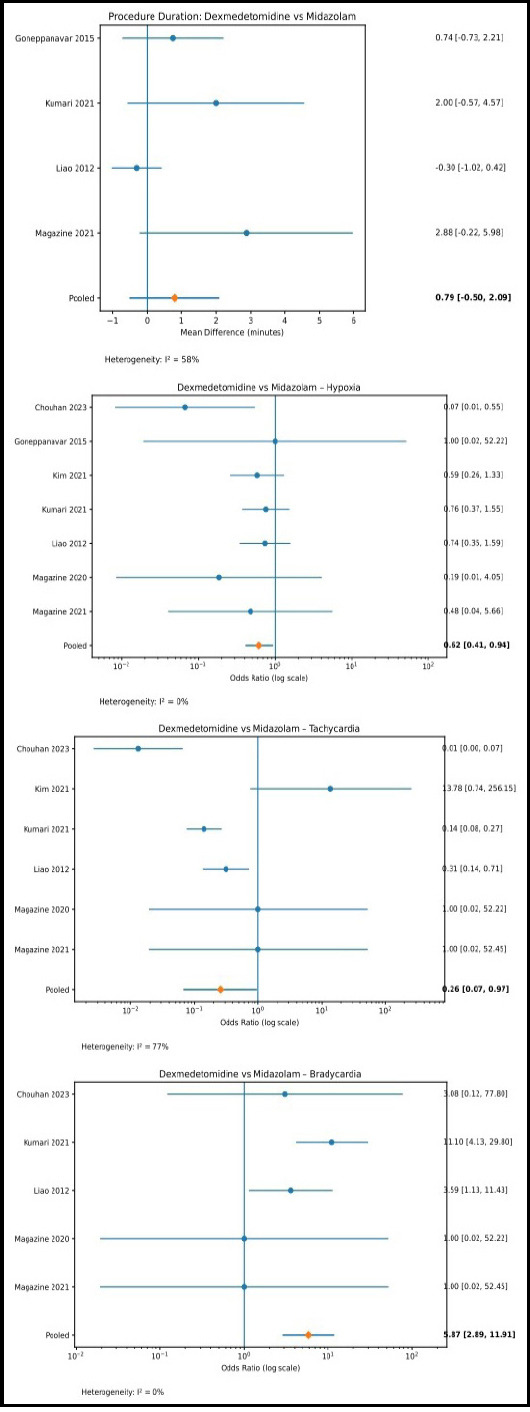
Meta-analysis for duration of bronchoscopy, hypoxia, tachycardia, bradycardia for Dexmedetomidine vs Midazolam.

**Supplementary Fig.3:**
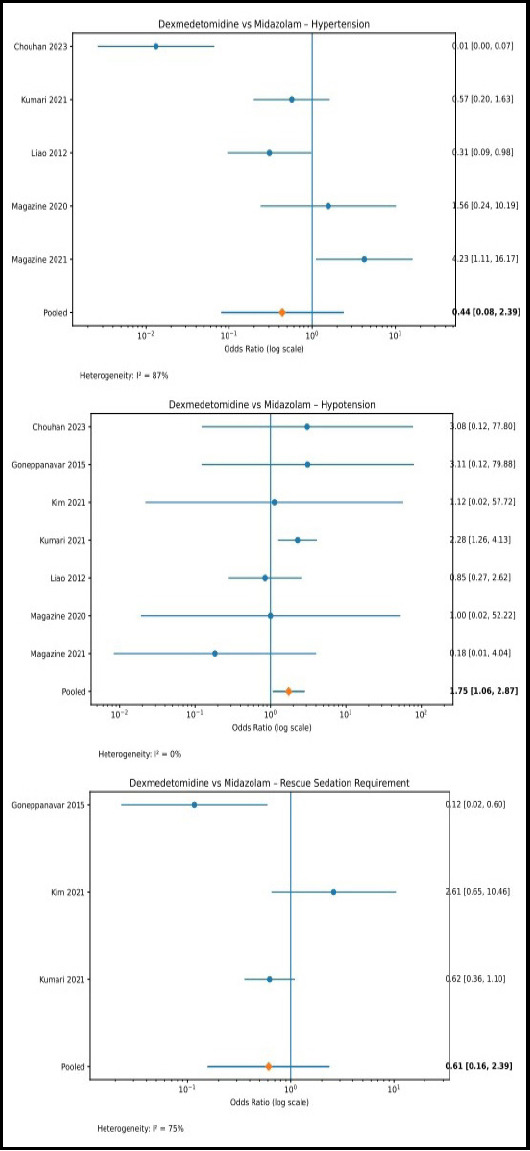
Meta-analysis for incidence of hypertension, hypotension, rescue sedation requirement during bronchoscopy with Dexmedetomidine vs Midazolam.

Pooled analysis is shown in Supplementary Fig.2 & 3. Meta-analysis showed no significant difference in bronchoscopy duration between dexmedetomidine and midazolam (MD 0.79 minutes, 95% CI −0.50 to 2.09, I² = 58%). The analysis also showed that dexmedetomidine significantly reduced the risk of hypoxia compared to midazolam (OR 0.62, 95% CI 0.41- 0.94, I² = 0%). Moreover, dexmedetomidine was associated with a lower incidence of tachycardia compared with midazolam (OR 0.26, 95% CI 0.07-0.97, I² = 77%). Pooled analysis also indicated a higher incidence of bradycardia in the dexmedetomidine group (OR 5.87, 95% CI 2.89- 11.91, I² = 0%), but there was no significant difference in the risk of hypertension (OR 0.44, 95% CI 0.08- 2.39, I² = 87%). However, dexmedetomidine was associated with a lower risk of hypotension (OR 0.62, 95% CI 0.41–0.94, I² = 0%). Few trials reported on the need for rescue sedation during bronchoscopy. The pooled analysis showed no significant difference between the two drugs (OR 0.61, 95% CI 0.16–2.39, I² = 75%). GRADE assessment showed “low” certainty of evidence for all outcomes, owing to bias in the included RCTs.

### Dexmedetomidine versus Propofol:

Procedural success was assessed only descriptively due to variations in definitions (Table-II). Chouhan et al.,^9^ showed that average composite score for procedural tolerance was in the ideal range in the dexmedetomidine group. Higher scores in the propofol group indicated poorer tolerance and cooperation compared with dexmedetomidine. Yuan et al.^18^ assessing cough and discomfort noted no statistically significant difference between dexmedetomidine and propofol. On the other hand, Lin et al.^20^ examining patient-reported wakefulness, sensory perception during sedation, and overall tolerance showed that patients receiving dexmedetomidine often reported being awake and perceiving sensations during the procedure, and were more likely to notice procedure-related symptoms compared to propofol.

**Supplementary Fig.4:**
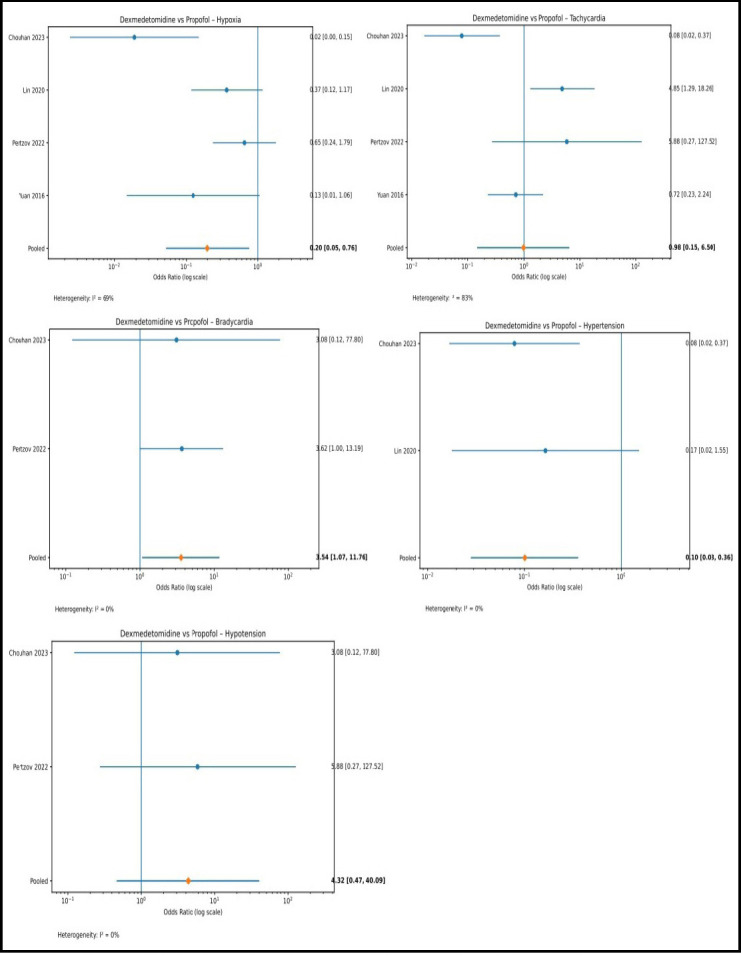
Meta-analysis for incidence of hypoxia, tachycardia, bradycardia, hypertension, and hypotension during bronchoscopy with Dexmedetomidine vs Propofol.

Pooled analysis is shown in Supplementary Fig.4. Meta-analysis showed a lower incidence of hypoxia with dexmedetomidine (OR 0.20, 95% CI 0.05–0.76; I² = 69%), but there was no significant difference in tachycardia rates between the two groups (OR 0.98, 95% CI 0.15–6.50; I² = 83%). A higher incidence of bradycardia was observed with dexmedetomidine (OR 3.54, 95% CI 1.07–11.76; I² = 0%). The analysis also showed a lower risk of hypertension (OR 0.10, 95% CI 0.03–0.36; I² = 0%) with dexmedetomidine, but there was no significant difference in hypotension rates between the two drugs (OR 4.32, 95% CI 0.47–40.09; I² = 0%). GRADE assessment showed “low” certainty of evidence for all outcomes, owing to bias in the included RCTs.

### Dexmedetomidine versus Remimazolam:

A descriptive analysis was performed for outcomes related to success (Table-II). Chen et al.,^10^ assessed sedation success based on achieving patient cooperation for intubation, the absence of rescue sedatives or local anaesthetics, and favourable intubation conditions. Using this criteria, both higher-dose remimazolam (0.093 mg/kg) and dexmedetomidine showed similarly high success rates as compared to lower-dose remimazolam. Dexmedetomidine also resulted in better intubation conditions compared to remimazolam. Xu et al.^11^ showed no significant difference between dexmedetomidine and remimazolam for intraoperative awareness. Zhou et al.^27^ reported a significantly lower interruption rate due to coughing or body movements in the remimazolam group compared to dexmedetomidine. Lastly, in Chen et al.,^25^ procedural success rates were high and similar across groups, with 94.5% in the remimazolam group and 91.8% in the dexmedetomidine group, without any significant difference.

**Supplementary Fig.5:**
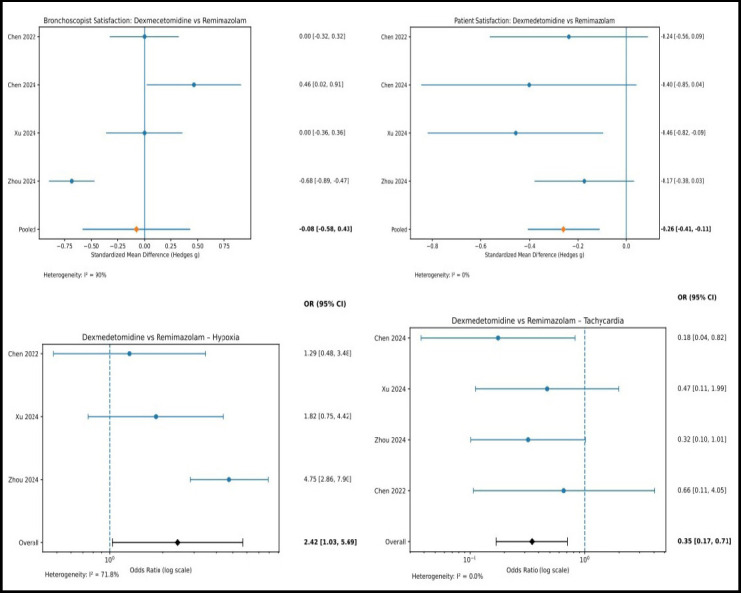
Meta-analysis for bronchoscopist & patient satisfaction score, hypoxia, tachycardia during bronchoscopy with Dexmedetomidine vs remimazolam.

**Supplementary Fig.6:**
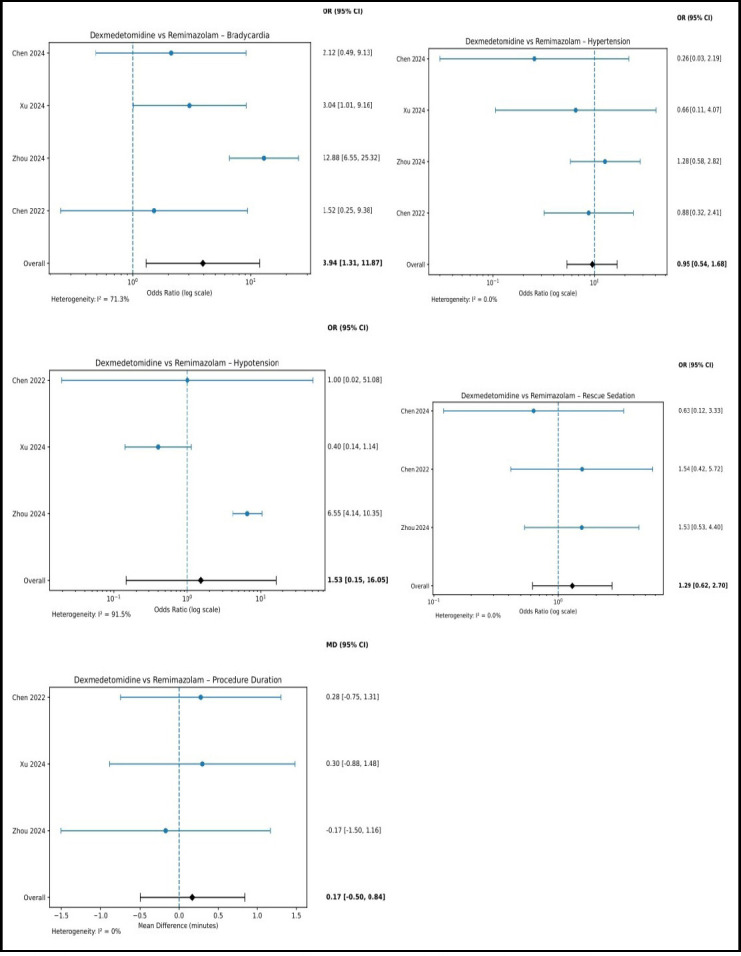
Meta-analysis for incidence of bradycardia, hypertension, hypotension, rescue sedation requirement, and procedure duration during bronchoscopy with Dexmedetomidine vs remimazolam.

Pooled analysis is shown in Supplementary Fig.5 & 6. Meta-analysis showed no difference in bronchoscopist satisfaction scores with dexmedetomidine compared to remimazolam (SMD −0.08, 95% CI −0.58 to 0.43; I² = 90%). Patient satisfaction scores were significantly lower with dexmedetomidine than with remimazolam (SMD −0.26, 95% CI −0.41 to −0.11; I² = 0%). The pooled analysis showed that dexmedetomidine was linked to a significantly higher risk of hypoxia compared to remimazolam (OR 2.42, 95% CI 1.03–5.69; I² = 72%). It also showed that dexmedetomidine had a significantly lower risk of tachycardia compared to remimazolam (OR 0.35, 95% CI: 0.17–0.71; I² = 0%). We observed that dexmedetomidine was associated with a significantly higher risk of bradycardia compared to remimazolam (OR 3.94, 95% CI: 1.31–11.87, I² = 71.3%). The meta-analysis found no significant difference in hypertension (OR 0.95, 95% CI: 0.54–1.68, I² = 0%) or hypotension (pooled OR = 1.53, 95% CI: 0.15–16.05, I² = 91.5%) between the two groups. No significant difference was found in rescue sedation requirements (OR = 1.29, 95% CI: 0.62–2.70; I² = 0%) or procedure duration between the two agents (MD = 0.17 minutes, 95% CI: −0.50 to 0.84; I² = 0%). GRADE assessment showed “low” certainty of evidence for all outcomes, owing to bias in the included RCTs.

### Dexmedetomidine versus opioids:

Literature comparing dexmedetomidine with other opioids is limited, so only descriptive analysis was performed. Ryu et al.^16^ compared dexmedetomidine-based sedation to remifentanil-based sedation during flexible bronchoscopy and noted that dexmedetomidine was associated with a significantly lower rate of oxygen desaturation. Hemodynamic adverse events such as tachycardia, hypotension, hypertension, and arrhythmias were more common with remifentanil, but without statistically significant differences. No bradycardia episodes occurred in either group. Procedure duration was similar between groups.

Riachy et al.^19^ compared dexmedetomidine-based sedation to alfentanil-based sedation, and showed that the overall procedural feasibility did not differ significantly between the two groups. Sedation depth assessed by the Nursing Instrument for the Communication of Sedation score was higher in the dexmedetomidine group before bronchoscopy. Hypoxia was more common with alfentanil, which also caused more heart rate changes, whereas dexmedetomidine was associated with more hypotensive episodes. No clinically significant bradycardia occurred. Procedure durations were similar across groups.

Zhang et al.^24^ compared dexmedetomidine with fentanyl for sedation during bronchoscopy. Dexmedetomidine resulted in fewer hypoxia and tachycardia incidents. It caused more hypotension and bradycardia. Hypertension and serious respiratory issues were similar between groups. Procedure duration was longer with dexmedetomidine, but procedural feasibility and patient-reported outcomes like discomfort, satisfaction, awareness, and willingness for repeat procedures were comparable.

## DISCUSSION

This systematic review and meta-analysis of 17 RCTs examined the effectiveness and safety of dexmedetomidine for adult bronchoscopy sedation compared to midazolam, propofol, remimazolam, and opioids. While success rates were high with all drugs, procedural success varied due to differing criteria used by the RCTs. Findings were mixed when comparing dexmedetomidine with midazolam and propofol, but outcomes seemed better with remimazolam. Quantitatively, dexmedetomidine reduced hypoxia and tachycardia versus midazolam, but increased bradycardia and reduced risk of hypotension. There was no difference in procedure length or rescue sedation. Compared to propofol, it similarly decreased hypoxia and hypertension but increased bradycardia, with comparable procedural feasibility. Compared to remimazolam, dexmedetomidine was linked to lower satisfaction and higher hypoxia risk, as well as increased bradycardia, but no consistent difference in other hemodynamic parameters, rescue sedation, or duration.

Adequate sedation is essential for a successful bronchoscopy to enhance patient comfort, reduce cough and anxiety, and create better conditions for the bronchoscopist. Inadequate sedation can cause patient discomfort, excessive movement, hypoxia, or early termination of the procedure. On the other hand, excessive sedation raises the risk of respiratory depression and hemodynamic issues. Finding the right balance is crucial.^3^ Traditionally, midazolam, propofol, and opioids have been routinely used for sedation in bronchoscopy.^5^,^6^,^30^ Drugs like midazolam offer reliable anxiolysis and anterograde amnesia, but they have a slower onset, longer recovery time, and limited pain relief, often causing coughs that require opioids. On the other hand, propofol allows for rapid onset and offset of sedation. It is usually combined with opioids but needs careful monitoring during use. The narrow margin between moderate and deep sedation levels, along with absence of pharmacologic antagonist, makes patients on higher doses vulnerable to respiratory depression and unstable blood pressure.^31^ Recently, dexmedetomidine has become an alternative sedative in bronchoscopy. Its high selectivity for α-adrenergic receptors and its unique action provide both sedation and pain relief while maintaining spontaneous breathing. It has been proven to be helpful for bronchoscopy and awake airway procedures.^7^,^12^ Nonetheless, use of dexmedetomidine in clinical practice should be based on strong evidence.

The present review compared dexmedetomidine with four major drugs/drug groups, namely midazolam, propofol, remimazolam and opioids. For procedural success and sedation adequacy, the majority of studies reported no difference between dexmedetomidine and midazolam, except for two studies^9^,^17^ that reported better outcomes with dexmedetomidine. On the contrary, comparison with propofol showed mixed results. One study reported better outcomes with dexmedetomidine,^9^ another with propofol^20^ and the third reported no difference.^18^ The qualitative analysis for dexmedetomidine vs remimazolam showed more consistent results. Most studies reported a tendency for better sedation with remimazolam. Importantly, adequate data were available for a meta-analysis of bronchoscopist and patient satisfaction scores, which showed consistent better outcomes with remimazolam as compared to dexmedetomidine. Only three RCTs were available comparing dexmedetomidine with opioids, and descriptive analysis showed that adequacy of sedation was similar between dexmedetomidine and other opioids.

This review’s findings expand upon the results of previous meta-analyses. The systematic review by Guo et al.,^12^ which included nine RCTs comparing dexmedetomidine with various control treatments, showed that dexmedetomidine reduces the risk of hypoxemia and tachycardia but increases the risk of bradycardia. They found no significant differences in hypertension, hypotension, or patient satisfaction. Our results are in agreement with their review, but substantially expand the evidence by including eight additional RCTs. We included newer studies comparing with remimazolam, and conducted separate analyses specific for each drug. Importantly, Guo et al.^12^ performed a subgroup analysis only for hypoxia. For all other outcomes, they combined various drugs in a single analysis, leading to inaccurate findings. Similarly, the more recent meta-analysis by Liang et al.,^6^ focused only on dexmedetomidine versus midazolam. They reported lower desaturation rates and a higher incidence of bradycardia with dexmedetomidine, but no significant difference in procedure duration. Our findings are similar to their results, but with a larger number of studies.

Our findings are also comparable with studies using dexmedetomidine for gastrointestinal endoscopic procedures. Tang et al.^30^ in a meta-analysis of 40 RCTs, showed that dexmedetomidine achieved sedation levels similar to other sedatives. There was no difference in Ramsay Sedation Scale scores or patient satisfaction between the groups. Dexmedetomidine was associated with a significantly lower risk of involuntary movements or gagging, a reduced need for additional sedatives, and higher satisfaction scores. Dexmedetomidine was also associated with a lower incidence of hypoxia and cough compared to other agents, with no significant difference in risk of hypotension. However, the risk of bradycardia was significantly increased in patients administered dexmedetomidine.

Cardio-respiratory safety is of utmost importance during sedation. Hypoxia is a critical concern during bronchoscopy, and dexmedetomidine demonstrated favourable results across most comparisons. In quantitative analyses, dexmedetomidine was consistently associated with a lower incidence of hypoxia than midazolam and propofol. This finding was also seen in the qualitative evidence wherein fewer desaturation episodes were noted in comparison with opioids. The minimal respiratory effects of dexmedetomidine are due to its high selectivity as an α2-adrenoceptor agonist. It primarily targets central pre- and postsynaptic α2-receptors in the locus coeruleus, leading to a distinctive sedative effect.^32^,^33^

In contrast, compared with remimazolam, dexmedetomidine was associated with a higher incidence of hypoxia, suggesting that the newer ultra–short–acting benzodiazepine may offer superior respiratory stability during bronchoscopy. However, the results must be interpreted with caution given the fact that only four RCTs compared dexmedetomidine with remimazolam, of which one^10^ reported no hypoxia events in any group. A recent meta-analysis by Tang et al.^34^ comparing dexmedetomidine with remimazolam for awake fibreoptic intubation has noted no difference in the success of intubation and sedation scores between the two drugs, but with reduced risk of hypoxia with dexmedetomidine.

Hemodynamic adverse events exhibited a more consistent pattern across different comparisons. Dexmedetomidine was linked to a reduced occurrence of tachycardia and hypertension, due to its sympatholytic effects. However, this advantage was counterbalanced by a notably increased risk of bradycardia and hypotension. The dose-dependent reduction in heart rate and blood pressure results from decreased endogenous catecholamine levels, peripheral vasoconstriction, sympatholysis, and baroreflex-mediated parasympathetic activation. These effects are associated with the activation of α2-adrenoceptor and imidazoline-preferring receptors in the ventrolateral medulla and solitarius nucleus tract.^32^,^33^ Clinicians should therefore be cautious of using dexmedetomidine in patients with severe sinus bradycardia or heart block. Avoiding rapid titration, lowering the initial loading dose, and prehydration are measures that can prevent these adverse events.^35^

### Limitations

A major limitation of the present review was the high heterogeneity noted for several outcomes, including hypertension (I²=87%), tachycardia (I²=77%), rescue sedation (I²=75%), and bronchoscopist satisfaction (I²=90%). We believe that this heterogeneity is likely due to differences in dexmedetomidine dosing protocols, comparator drug regimens, concomitant use of adjuncts such as lidocaine or midazolam, variation in bronchoscopy type, and differences in patient risk profiles across included trials. The methodological differences between the studies were too vast and the total number of studies for each comparison were too scarce to explore the sources of heterogeneity. This precluded a formal subgroup analyses or meta-regression in this review. Given the high heterogeneity, the results must be interpreted with caution.

First, while procedural success and sedation adequacy are key outcomes, a quantitative synthesis was unfeasible due to inconsistent definitions and reporting across studies, limiting analysis to narrative description. Sedation success was assessed as composite procedural tolerance scores in some studies, while others defined success as the absence of rescue sedation or favorable intubation conditions. Several other studies did not formally define the outcome. This limitation precluded quantitative synthesis of this core outcome. Second, pooling of outcomes such as cough, sedation level, and rescue medication was again limited by differences in outcome definitions, grading, assessment timing, and data reporting. Satisfaction scores and rescue sedation requirements could not be combined across all comparators due to limited data. Many studies had small sample sizes and were single-center, possibly affecting generalizability. Furthermore, the predominance of Asian studies suggests limited applicability to Western populations. It is also important to note that the three RCTs comparing dexmedetomidine with opioids used different agents (remifentanil, alfentanil, and fentanyl). These differ substantially in onset, duration of action, and potency. While pooling data from these studies was not attempted, and the descriptive findings should be interpreted with caution given this clinical heterogeneity.

## CONCLUSIONS

Evidence shows that dexmedetomidine can be an effective sedative option for adult bronchoscopy, with sedation levels that seem equivalent to those with midazolam and propofol. Dexmedetomidine appears to be associated with reduced hypoxia and sympathetic responses when compared with midazolam and propofol, but increases the risk of bradycardia. In contrast, when compared with remimazolam, dexmedetomidine was associated with less favourable sedation and satisfaction outcomes and a higher incidence of hypoxia, however, evidence was generated from a small number of studies limiting strong conclusions. All of the review results are from a limited number of studies with heterogeneous protocols. Further robust multi-centric RCTs are needed to improve the quality of evidence.

### Authors’ contributions:

**XH:** Literature search, study design and manuscript writing.

**JL, LZ, YD, HS** and **JZ:** data collection, data analysis and interpretation. Critical Review.

**XH:** Manuscript revision and validation and is responsible for the integrity of the study. All authors have read and approved the final manuscript.
